# Exploring the effects of structure and melting on sweetness in additively manufactured chocolate

**DOI:** 10.1038/s41598-024-58838-6

**Published:** 2024-04-09

**Authors:** Johannes Burkard, Lucas Kohler, Sophia Caciagli, Nicolas Herren, Mark Kozamernik, Saskia Mantovani, Erich J. Windhab, Christoph Denkel

**Affiliations:** 1 ETH Zurich, Institute of Food, Nutrition and Health, 8092 Zürich, Switzerland; 2 School of Agricultural, Forest and Food Sciences HAFL, Food Science and Management, Bern University of Applied Sciences, 3052 Zollikofen, Switzerland

**Keywords:** Health occupations, Engineering, Materials science

## Abstract

In view of the health concerns associated with high sugar intake, this study investigates methods to enhance sweetness perception in chocolate without increasing its sugar content. Using additive manufacturing, chocolate structures were created from masses with varying sugar and fat compositions, where hazelnut oil served as a partial cocoa butter replacement. The study found that while variations in sugar content minimally affected the physical properties of the chocolate masses, hazelnut oil significantly modified melting behavior and consumption time. Chocolate masses with higher hazelnut oil content but similar sugar content exhibited a 24% increase in sweetness perception, likely due to accelerated tastant (i.e., sucrose) release into saliva. Multiphase structures, designated as *layered*, *cube-in-cube*, and *sandwich* structures, exhibited less sensory differences compared to the homogeneous control. Nonetheless, structures with hazelnut oil-rich outer layers resulted in an 11% increase in sweetness perception, even without sugar gradients. This suggests that tastant release plays a more critical role than structural complexity in modifying sweetness perception. This research highlights the efficacy of simpler multiphase structures, such as *sandwich* designs, which offer sensory enhancements comparable to those of more complex designs but with reduced manufacturing effort, thus providing viable options for industrial-scale production.

## Introduction

The marked increase in life expectancy, coupled with lifestyle changes in Western countries, have led to significant increases in obesity and non-communicable diseases (NCDs)^1^. Although added sugar consumption has declined in recent years^2–5^, current intake still exceeds the recommended 5 to 10% of daily energy consumption^6^. This discrepancy underscores the need for low-sugar food alternatives and compels food manufacturers to explore effective sugar reduction strategies^7,8^.

However, sugar replacement in processed foods is challenging because it contributes to both taste and texture, as demonstrated in products such as ice cream^9^, cookies^10^ or chocolate^11^. The predominant strategy for reducing dietary sugar involves replacing sugar with non-nutritive sweeteners (such as sucralose, aspartame, acesulfame potassium, saccharin, stevia, thaumatin or monk fruit)^8^, although limited by their bitter or metallic aftertaste^12–15^. An emerging alternative which dispenses with non-nutritive sweeteners focuses on the modification of food structure^8,16–18^. It seeks to alter taste perception by applying a concept known as “pulsatile stimulation”. This technique uses tastant (i.e., sucrose) gradients within samples to discontinuously stimulate taste receptors and enhance overall sweetness perception compared to a homogeneous control. This enhancement has been validated in a variety of matrices, including sweet solutions^19–21^, multi-layered gels^16–18,22^, jet-printed chocolate surfaces^23^, or 3D-printed chocolate^24^. For example, Kistler et al.^22^ found that hydrocolloid structures with a sucrose gradient, created using fused deposition modeling (FDM), were perceived as up to 30% sweeter than samples with homogeneously distributed sugar.

In this context, we used FDM to manufacture chocolate structures with inhomogeneous sucrose distribution, aiming to increase the sweetness perception. Critical physical properties, such as flow behavior and particle size distribution, were maintained uniform in both the low-sugar and the high-sugar chocolate masses, which served as masses for the multiphase structures. This methodological choice allowed us to focus how the spatial sugar arrangement influences taste perception in complex fat-based structures, while reducing the influence of confounding factors. In a first set of experiments, we prepared five multiphase structures from the two chocolate masses, varying from *layered* to *cube-in-cube* designs, which were then sensorially evaluated by a trained panel (N = 8) against a homogeneous control of similar total sugar content. A follow-up set of experiments comprised the design of structures from chocolate masses that differed in both sugar content and melting speed, achieved by partially substituting cocoa butter with hazelnut oil. Extensive analytical and sensory analyses were carried out on all the chocolate masses. The final stage involved the sensory evaluation of six *sandwich* structures, designs consisting of either one or three inner layers with distinct sugar and fat compositions and two identical top and bottom layers. The *sandwich* structures were evaluated by the same panel, using comparative and time-intensity methods (N = 8).

Our experiments explored the influence of mass composition, structural design, and manufacturing effort on the perceived sweetness of chocolate structures, all while maintaining controlled physical properties. This study aimed to improve our understanding of how sweetness perception in chocolate can be adjusted by additive manufacturing and relate it to pulsatile stimulation hypotheses.

## Results and discussion

### Chocolate mass analysis

#### Instrumental analysis

To evaluate the effect of inhomogeneous sucrose distribution on sweetness perception within multiphase structures, it was critical to ensure that all chocolate masses had comparable techno-functional properties. This uniformity should minimize the influence of somatosensory factors on taste perception^25^. To achieve this uniformity, especially in samples with different sugar content, sugar was partially replaced with a mixture of inulin and polydextrose, both known for their bulking properties^26,27^.

**Figure 1:**
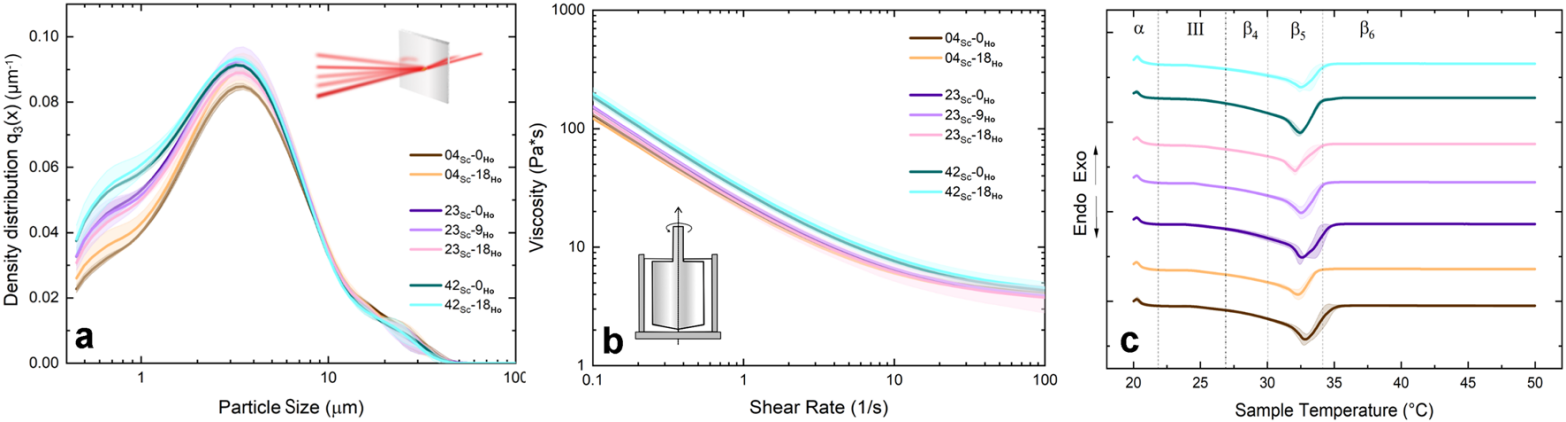
Instrumental characterization of chocolate masses with different sugar (*Sc*) and hazelnut oil (*Ho*) contents. (**a**) Particle size distribution, (**b**) rheological flow curves, (**c**) differential scanning calorimetry (DSC) curves: the dashed lines delimit the distinct melting domains associated with different cocoa butter polymorphs^28^. In all three panels (**a**), (**b**) and (**c**) the standard deviation of each sample is depicted as the shaded area around the mean.

After seven hours of grinding, all chocolate masses exhibited a unimodal size distribution pattern with a d$_{90}$ value below $30\, \upmu \hbox {m}$, which is essential to avoid a gritty texture^29,30^ (Fig. 1a). The particle size distribution was not significantly influenced by the sugar (F(2,7)[d$_{90}$] = 0.34, NS) or hazelnut oil (*Ho*) (F(2,7)[d$_{90}$] = 0.24, NS) content.

The flow curves in Fig. 1b demonstrate the non-Newtonian pseudoplastic behavior typical of chocolate^31–33^. Analysis of the viscosity at shear rates of 10, 20, and 50 s$^{-1}$ showed a linear relationship with the sugar content. Notably, significant differences were found only at a shear rate of 10 s$^{-1}$, as detailed in Supplementary Information 7. This is in contrast to previous studies by Aidoo et al.^27,34^, which reported an increased bulk viscosity for chocolates rich in polydextrose/inulin, due to their lower density and the resulting larger surface area that had to be coated with cocoa butter. In our study, the contrasting results may be explained by the irregular fracture patterns of crystalline sugar and amorphous polydextrose/inulin particles resulting from our small-scale grinding process. While this process predominantly affected particle shape, hazelnut oil (e.g., F(2,7)[$\eta _{10}$] = 2.07, NS) and sugar content only had minimal effects on particle size distribution and flow behavior.

A consistent endothermic transition between $25.2\,^{\circ }\hbox {C}$ and $34.9\,^{\circ }\hbox {C}$ was observed in all the samples detailed in Fig. 1c, with melting peaks around $32\,^{\circ }\hbox {C}$ to $33\,^{\circ }\hbox {C}$. The triglyceride composition of hazelnut oil closely mirrors that of cocoa butter, particularly in the chain length^35^. This similarity, combined with the moderate hazelnut oil levels used, indicates a uniform crystallization behavior in all chocolate masses, regardless of hazelnut oil content^36^. While cocoa butter substitution significantly affected several calorimetric characteristics, the most pronounced effect was found for melting enthalpy, which decreased from about 44-45 J/g in pure chocolate to 35-37 J/g in blends with 18 %w hazelnut oil/total fat. However, the sugar content (e.g., F(2,7)[melting enthalpy] = 1.93, NS) showed no significant effect on the melting properties, as detailed in Supplementary Information 7.

#### Sensory analysis

**Figure 2:**
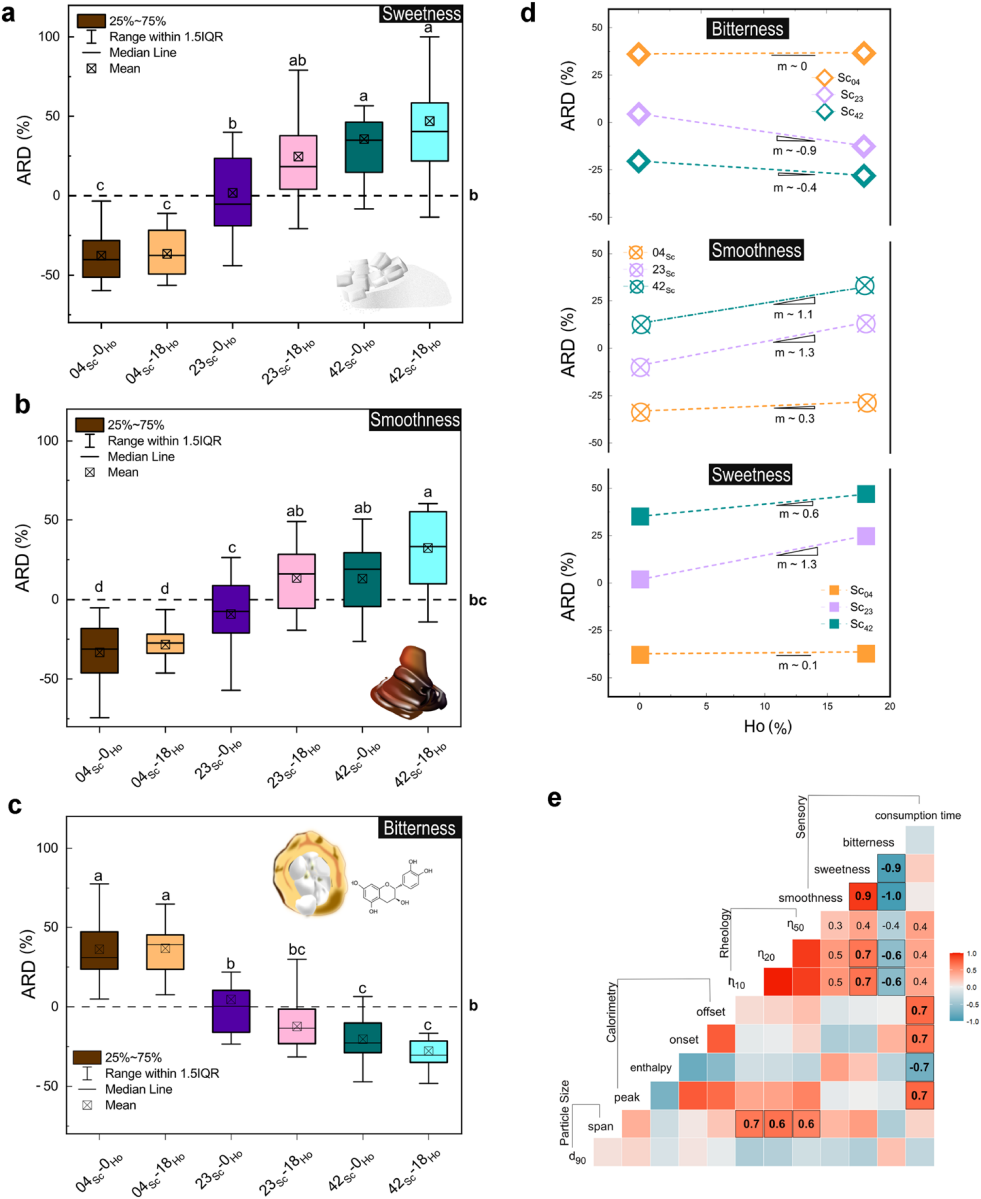
Sensory evaluation of chocolate masses with different contents of hazelnut oil (*Ho*) and sucrose (*Sc*). Panels (**a**), (**b**) and (**c**) show the average relative difference from control (ARD) for sweetness, smoothness, and bitterness perception, respectively. Panel (**d**) illustrates the interplay between sugar (*Sc*) and hazelnut oil (*Ho*) content across the three sensory attributes. Here, *m* represents the slope of the relationship between *Ho* content and perceptual ARD for each attribute and sugar level, where a positive *m* indicates a positive relationship and vice versa. ARD values with matching subscripts are not significantly different (p > 0.05). Panel (**e**) provides a correlation matrix comparing sensory ratings with instrumental characteristics of the chocolate masses, highlighting both significant (bold and framed) and relevant correlations for clarity.

The expected correlation between the sugar content and sensory sweetness was confirmed (F(2,98)[*Sc*] = 79, *p* < 2e-16). However, a more intriguing finding was the significant effect of hazelnut oil content on sweetness perception (F(2,98)[*Ho*] = 4.2, p $=$ 0.01). Notably, the average relative difference of sweetness from the homogeneous control (hereafter referred to as ARDs) for the samples *42*$_{Sc}$-*0*$_{Ho}$ and *23*$_{Sc}$-*18*$_{Ho}$ were comparable, despite the former having nearly twice the sugar content (see Fig. 2a). Blending cocoa butter with hazelnut oil lowered the melting enthalpy as indicated in the previous chapter, reduced the consumption time (see Fig. 2e, R$^{2}$ = -0.7, p = 0.002), and potentially increased the availability of tastants in saliva.

To isolate the physicochemical effect of hazelnut oil on sucrose release, two matrices with identical sugar content (30 %w/w) but different fat compositions were prepared. In one, the fat component was all cocoa butter (70 %w/w), while in the other, 18 %w/w of the cocoa butter was replaced by hazelnut oil, representing the ratio used in the *23*$_{Sc}$-*18*$_{Ho}$ sample. The panelists licked both samples for 45 seconds, after which the samples were expectorated and their soluble content was quantified by refractometry (see Supplementary Information 9). Our results showed that salivary secretion (g saliva/g sample) was not significantly different between the two samples, nor did it contribute to the differences in soluble solids content (F(1,33) = 3.58, NS) (see Supplementary Fig. S5c). While previous research has shown that taste influences salivary secretion^37^, it is likely that the compositional differences between the two samples were too subtle. Despite individual differences in Brix measurements among the panelists (see Supplementary Fig. S5a), a clear trend emerged: samples containing hazelnut oil had higher soluble solids contents (F(1,30) = 15.31, *p* < 0.001), on average more than 2 Brix higher than those made with pure cocoa butter. This enhancement is probably due to the increased availability of sucrose, a consequence of the differences in melting enthalpy between hazelnut oil and cocoa butter, which allows a more efficient release of sucrose from the fat matrix. These results suggest that the incorporation of hazelnut oil enhances sweetness perception.

Furthermore, the strong positive correlation observed between sensory sweetness and smoothness in our chocolate samples, as illustrated in Fig. 2b+e (R$^{2}$ = 0.9, *p* < 10e-6), highlights the importance of understanding the physical factors contributing to sensory smoothness. Andablo-Reyes et al.^38^ suggested that the perception of smoothness is rooted in oral-tribological interactions, shifting the focus from viscosity to friction as a key determinant. Our tribology data, presented in Supplementary Information 10, shows that chocolate masses with higher sugar content exhibited increased coefficients of friction. This finding challenges the traditional belief of an inverse relationship between smoothness and friction^39^ and may be explained by the aggregation of sugar particles at low shear rates. The lack of a clear inverse correlation between both the friction coefficient and viscosity, and sensory smoothness, may indicate that the perceived textural differences in our chocolate samples may be influenced by perceptual factors rather than their physicochemical properties.

Sensory bitterness (ARDs) was most pronounced in the low sugar content and high cocoa solids samples (see Fig 2c). This increased bitterness can be attributed to alkaloids such as theobromine and caffeine, as well as flavan-3-ols found in cocoa solids, which are known for their bitter taste^40^. An inverse relationship was observed between both sugar (F(2,98) = 112.72, *p* < 2e-16) and hazelnut oil content (F(2,98) = 0.23, *p* < 0.02) and bitterness perception. This suggests that hazelnut oil can effectively mask the sensory bitterness^41^. This effect was particularly noticeable in chocolate masses with intermediate levels of sugar, as shown by a pronounced slope between hazelnut oil and sugar in Fig. 2d. At the extremes of sugar content, either the cocoa solids or sugar levels were too high for hazelnut oil to effectively suppress bitterness.

These findings indicate the possibility to modify multimodal perception in chocolate by up to 24% without changing the sugar content. As depicted in Fig. 2d, incorporating hazelnut oil not only increases sweetness and smoothness but also decreases sensory bitterness. This effect is most notable in samples of medium sugar content, as evidenced by the steep slopes (*m*) in Fig. 2d. Building on these results, it was of interest to explore to what extent these sensory profiles, especially sensory sweetness, are retained or altered when structuring multiple chocolate masses together by FDM.

### Multiphase structure analysis and pulsatile stimulation

**Figure 3:**
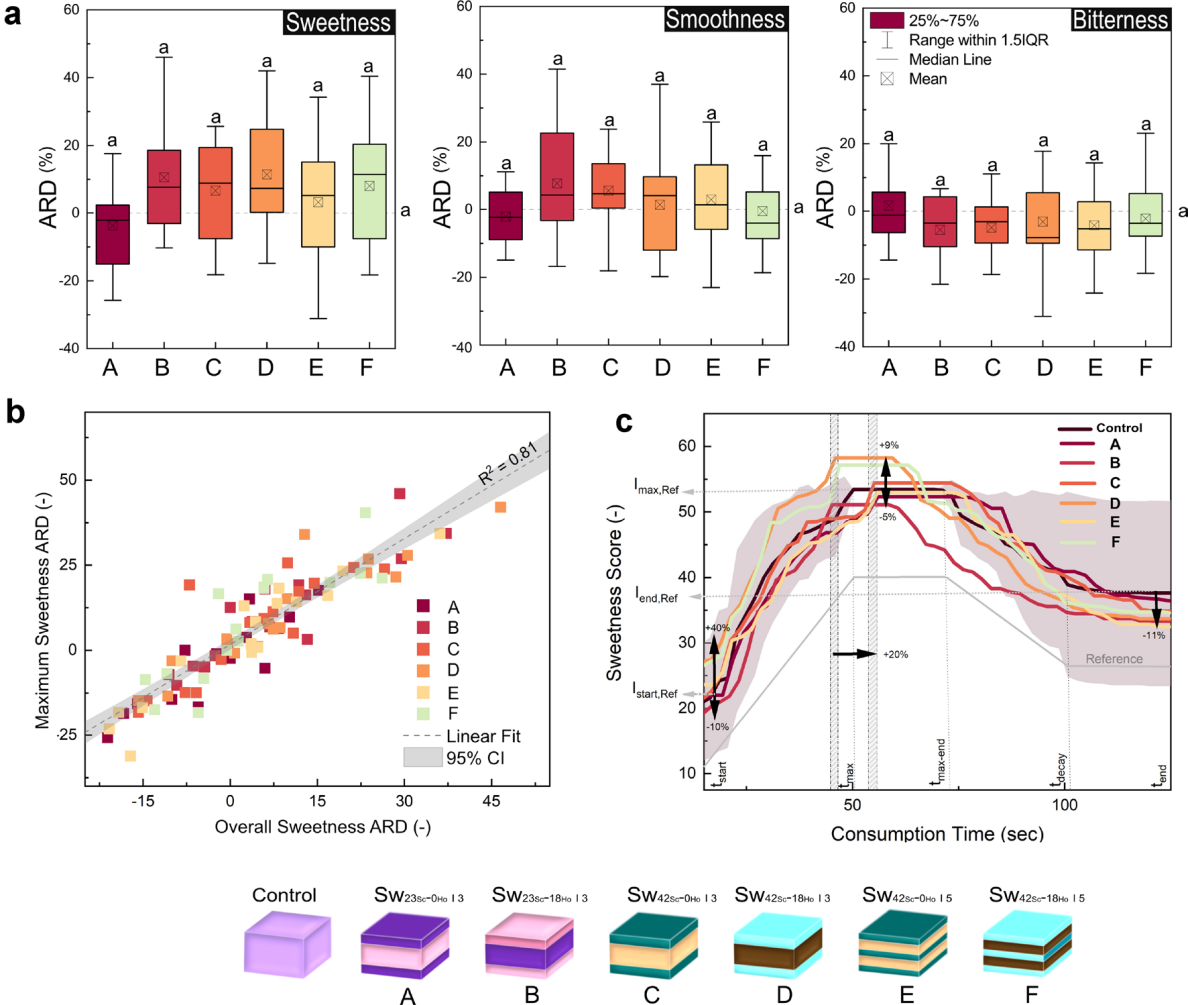
(**a**) Evaluation of the sensory attributes of multiphase chocolate structures during experimental set 2 (A = *Sw*${_{23}}{_{Sc}}$-*0*$_{Ho}$*I3*, B = *Sw*${_{23}}{_{Sc}}$-*18*$_{Ho}$*I3*, C = *Sw*${_{42}}{_{Sc}}$-*0*$_{Ho}$*I3*, D = *Sw*${_{42}}{_{Sc}}$-*18*$_{Ho}$*I3*, E = *Sw*${_{42}}{_{Sc}}$-*0*$_{Ho}$*I5*, F = *Sw*${_{42}}{_{Sc}}$-*18*$_{Ho}$*I5*), with results presented as average relative differences in perceived sweetness, smoothness, and bitterness from the control (ARD). ARD values with the same subscript are statistically indistinguishable (*p* > 0.05). Panel (**b**) shows the correlation between the overall and maximum sweetness ARD, including a linear fit. Panel (**c**) summarizes the temporal evolution of sweetness perception as assessed by time-intensity measurements. The arrows at the beginning (I$_{start,Ref}$), at the maximum (I$_{max,Ref}$), and at the end (I$_{end,Ref}$) of the plot indicate the dimension of the deviations from the homogeneous reference at different time points. The time regime is divided into three stages: onset, maximum plateau, and decay. It is shown for the reference sample (light gray line below the sample time-intensity curves). Furthermore, the horizontal arrow between the two vertical hatched areas indicates the delay of the peak intensity onset caused by the substitution of *Ho* in the tongue proximal layer. The standard deviation of the reference sample is shown (shaded area) to illustrate the variability in sensory perception.

The first set of experiments focused on modifying sweetness perception by inhomogeneous sucrose distribution. Exposure to sucrose was critical for eliciting a sweetness response, which was particularly pronounced in multiphase structures with high sucrose content in the outer layers, which facilitated the immediate release of sucrose upon melting. In particular, the structure *CiC*${_{42}}{_{Sc}}$-*0*$_{Ho}$ *I 2* was perceived as 31% sweeter than *CiC*${_{04}}{_{Sc}}$-*0*$_{Ho}$ *I 2*, as indicated by their ARD at *t*$_{start}$, seen in Supplementary Fig. S4. This observation was further supported by the time-intensity data from the second set of experiments, detailed in Fig. 3c. While this disparity, particularly between *Sw*${_{42}}{_{Sc}}$-*0*$_{Ho}$ *I 3* and *Sw*${_{42}}{_{Sc}}$-*18*$_{Ho}$ *I 3*, was pronounced at the onset of consumption (*t*$_{start}$), it gradually diminished as consumption progressed.

In contrast to chocolate masses, multiphase structures did not show a strong correlation between sweetness perception and smoothness or bitterness (Fig. 3a). This observation extends to both maximum and overall sensory perception, where a consistent correlation is shown across all attributes, with sweetness perception shown as an example in Fig. 3b. In comparison, Khemcheevakul et al.^24^, who also used FDM to create multilayer chocolate rings, managed to reduce sugar content by 19% in their chocolate multiphase structures without compromising sweetness or consumer preference. However, they did not extensively control the textural differences between high-sugar and low-sugar chocolate masses, which are critical in modulating taste perception at a physicochemical level. This aspect of the influence of texture on taste perception is further emphasized by Mosca et al.^17^, who showed that altering fracture properties in multi-layered hydrogels can manipulate taste perception by changing the total surface area, a critical factor in taste perception.

Further, it is well documented that sweetness perception can be influenced not only multimodally, but also by the pattern in which taste stimuli are presented^16–22,24,42,43^. Specifically, a discontinuous presentation of sweet tastants has been shown to modify the perceived intensity. However, our research suggests that changes in fat composition alone, even without changing sweetness levels, can enhance sweetness perception. For example, the ARD in sweetness perception for the structure *L*${_{23}}{_{Sc}}$-*18*$_{Ho}$ *I 3* was 15% higher than that of *L*${_{23}}{_{Sc}}$-*0*$_{Ho}$ *I 3*. This finding suggests the involvement of multiple mechanisms, colloquially referred to as “pulsatile stimulation”.

One explanation is inspired by the “Brück-Bartley” effect observed in vision science, where a flickering light is perceived as brighter than a steady light^44^. This analogy is applied to taste, where rodents exposed to high-frequency pulsed taste stimuli have been reported to experience a greater taste magnitude^45,46^, but is unlikely to apply to semi-solid foods^19^. An alternative hypothesis is that alternating sucrose concentrations could prevent taste adaptation^47–49^. However, our experiments comparing the structures (*L*${_{42}}{_{Sc}}$-*0*$_{Ho}$ *I 2* and *L*${_{42}}{_{Sc}}$-0$_{Ho}$ *I 12*) with different numbers of layers (the transfer from one layer to another being supposed to account for a pulse) did not show increased sweetness perception with more layers. This suggests that adaptation occurred regardless of pulse frequency, or because layers of different composition merged into a single perception at later stages. More compellingly, research suggests that expectation, shaped by textual or direct taste cues, significantly influences taste perception^50–52^. A study by Wilton et al.^53^ found that subjects rated low-sweet solutions as much sweeter when they were presented with conflicting cues, highlighting the power of expectation. Our study supports this, suggesting that the initial sweet stimulus sets a contextual framework that influences overall taste judgments. This aligns with the concept of cognitive bias, where the first impression of sweetness is preconsciously stored and integrated into overall perception^54^. In summary, our findings indicate that the variation in sweetness perception in semi-solid foods through pulsatile stimulation is largely due to this initial contextual shift.

### Synthesis and industrial relevance

**Figure 4:**
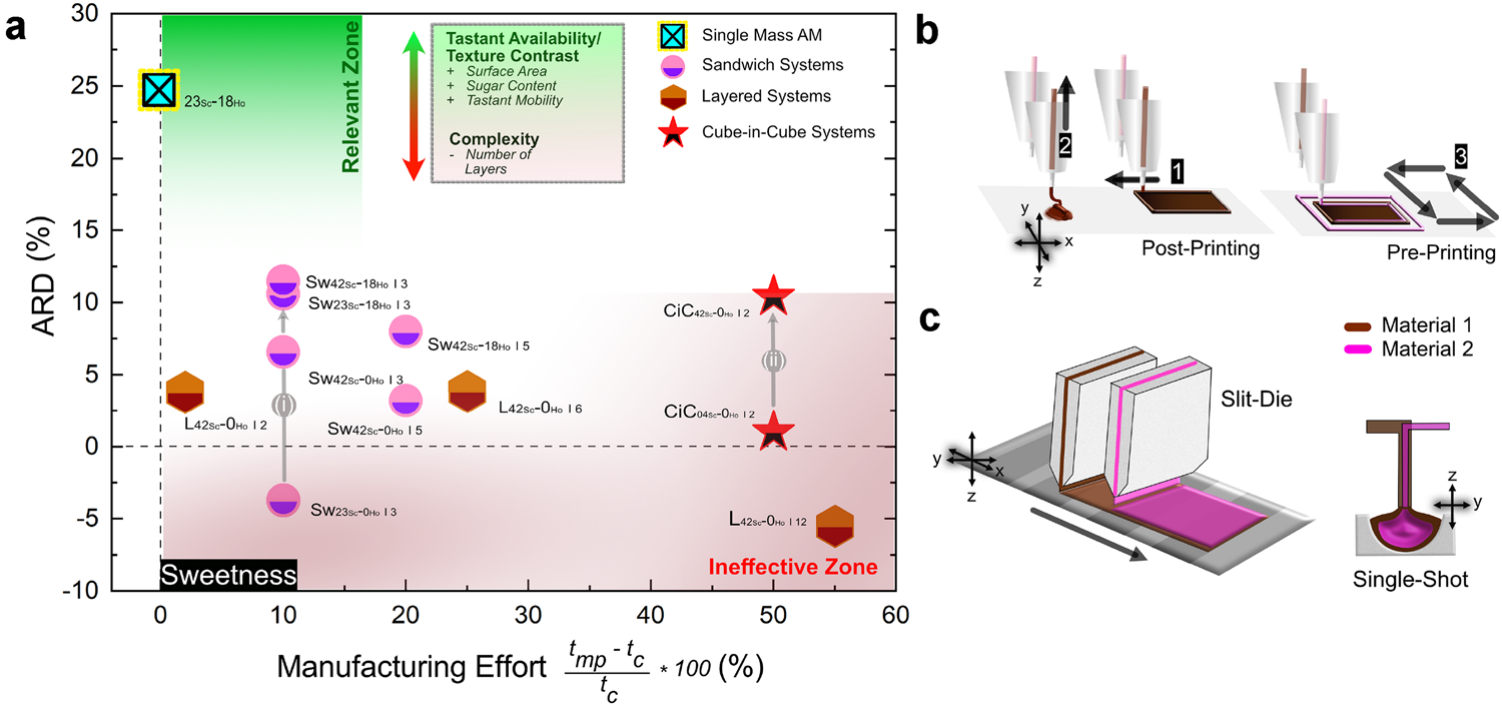
(**a**) Summary of the average relative difference from the control (ARD) for sweetness perception in both experiments (y-axis). The x-axis represents the manufacturing effort, calculated as the relative difference in manufacturing time between each multiphase structure (*t*$_{mp}$) and a single-phase homogeneous control (*t*$_{c}$) when printed by FDM. For reference, the chocolate mass *23*$_{Sc}$-*18*$_{Ho}$ is added and positioned at 0 on the x-axis for comparison. Although this sample was manufactured by molding (subtractive manufacturing), its position represents a scenario where the sample is made by FDM. A box on the graph summarizes the factors influencing sweetness perception: positive features are marked in green, negative ones in red. The “relevant zone” (green area) indicates an empirically defined region of reduced manufacturing effort and increased taste enhancement, in contrast to the “ineffective zone” (red area). Arrows (i) and (ii) emphasize the importance of the initial phase in sweetness perception, showing similar trends across different structures: (i) for *layered* samples and (ii) for *cube-in-cube* structures. (**b**) Steps of the FDM printing process, divided into pre-printing (steps 1-2) and post-printing (steps 3-4), which are crucial for precise material deposition. (**c**) Two possible industrial setups for fabricating multi-phase structures: slit-die strand extrusion and single-shot ejection.

Manufacturing effort in additive manufacturing (AM), defined here as the relative difference in time required to create a multiphase structure by AM compared to a single-phase homogeneous control, is a significant barrier to the adoption of AM in the food industry^55^. Our study highlights this challenge, with production time already exceeding four minutes for a single-phase cube (referred to as 0% on the x-axis in Fig. 4a). This increase is primarily due to the complexities of handling chocolate: as a non-Newtonian printing medium it requires careful management of crystallization kinetics and printing speed to achieve precise 3D structures. These factors are critical to ensure sufficient yield stress for layer stacking and to prevent shape distortion^56^.

This is particularly challenging for multiphase structures, such as *cube-in-cube* designs, where print tool transitions require additional retraction and extrusion steps to prevent dripping and nozzle blockage^57–59^, as shown in Fig. 4a+b. In contrast, layer-by-layer deposition techniques, which are suitable for creating *sandwich* structures, could significantly reduce the manufacturing effort. These structures, especially those with outer layers rich in sugar and/or hazelnut oil, ensure a sweetness perception comparable to more complex designs, but are simpler to manufacture, making them more viable for industrial-scale production, as shown in Fig. 4a. Specifically, *sandwich* structures, such as *Sw*${_{42}}{_{Sc}}$-*18*$_{Ho}$ *I 3*, not only have increased tastant availability due to sugar and hazelnut oil enriched contact areas, but also can be efficiently produced using parallel slit-die extruders (Fig. 4c). While *cube-in-cube* structures could be manufactured by the same technology, they would require more advanced slit-die configurations. However, these would not be able to match the speed of single-shot processes (Fig. 4c), which are the optimal scenario for products containing only spherical objects with a sweet outer layer.

Despite these speed limitations, AM processes offer significantly greater production flexibility and the ability to create texturally more complex structures. This flexibility is a key advantage of AM, allowing for a wider range of product designs and textures that can enhance the consumer experience.

## Conclusions

This study investigated the interplay between sweetness perception and the spatial distribution of tastants (sucrose) and fat phase (hazelnut oil fraction) in chocolate structures. Our results reveal that, while variations in sugar content minimally affected physical properties, partially substituting cocoa butter with hazelnut oil markedly decreased both melting enthalpy and consumption time. This change likely accelerated the release of sucrose into the saliva, a theory corroborated by in vivo expectoration tests. These tests demonstrated that the increased tastant release could account for the observed increase in sweetness perception, which reached up to 24% in samples containing hazelnut oil. Interestingly, our study could not confirm the generally recognized inverse correlation between the rheological and tribological properties of chocolate and its perceived smoothness, suggesting that smoothness may be integrated with sweetness perception at a cognitive level.

For multiphase structures, sensory differences were less pronounced. Experiments indicated that sweetness perception was enhanced in *cube-in-cube* structures with high sugar content in the outer layers, underscoring the importance of initial tastant release in setting a perceptual baseline for overall sweetness perception. However, increasing the structural complexity, such as adding more layers with different sugar content, did not enhance sweetness perception, suggesting that structural intricacy may have a limited effect on altering sweetness perception. *Sandwich* designs with identical top and bottom layers achieved sweetness perception levels comparable to the *cube-in-cube* structures. Remarkably, *sandwich* structures featuring only hazelnut oil gradients were perceived as equally sweet as those with sugar gradients, challenging the prevailing assumption about the necessity of sugar gradients for sweetness enhancement.

Our research suggests that in solid, fat-continuous systems like chocolate, the effect of sugar gradients on sweetness perception is small, particularly when the analytical textural differences within the samples are small. These subtle effects are further diminished in complex designs or when initial contact surfaces have contrasting sugar gradients that neutralize each other. In addition, the manufacturing effort is a significant barrier, especially for *cube-in-cube* designs, due to the numerous printer-tool transitions that require additional effort to prevent dripping or nozzle clogging. Therefore, future research should prioritize the development of simpler, yet effective, multiphase structures. These should be based on formulations that optimize tastant release and exhibit pronounced analytical and sensory texture gradients. Such advances will change the sensory perception of food products like chocolate, both physicochemically and perceptually.

## Methods

### Chocolate mass manufacturing

**Figure 5:**
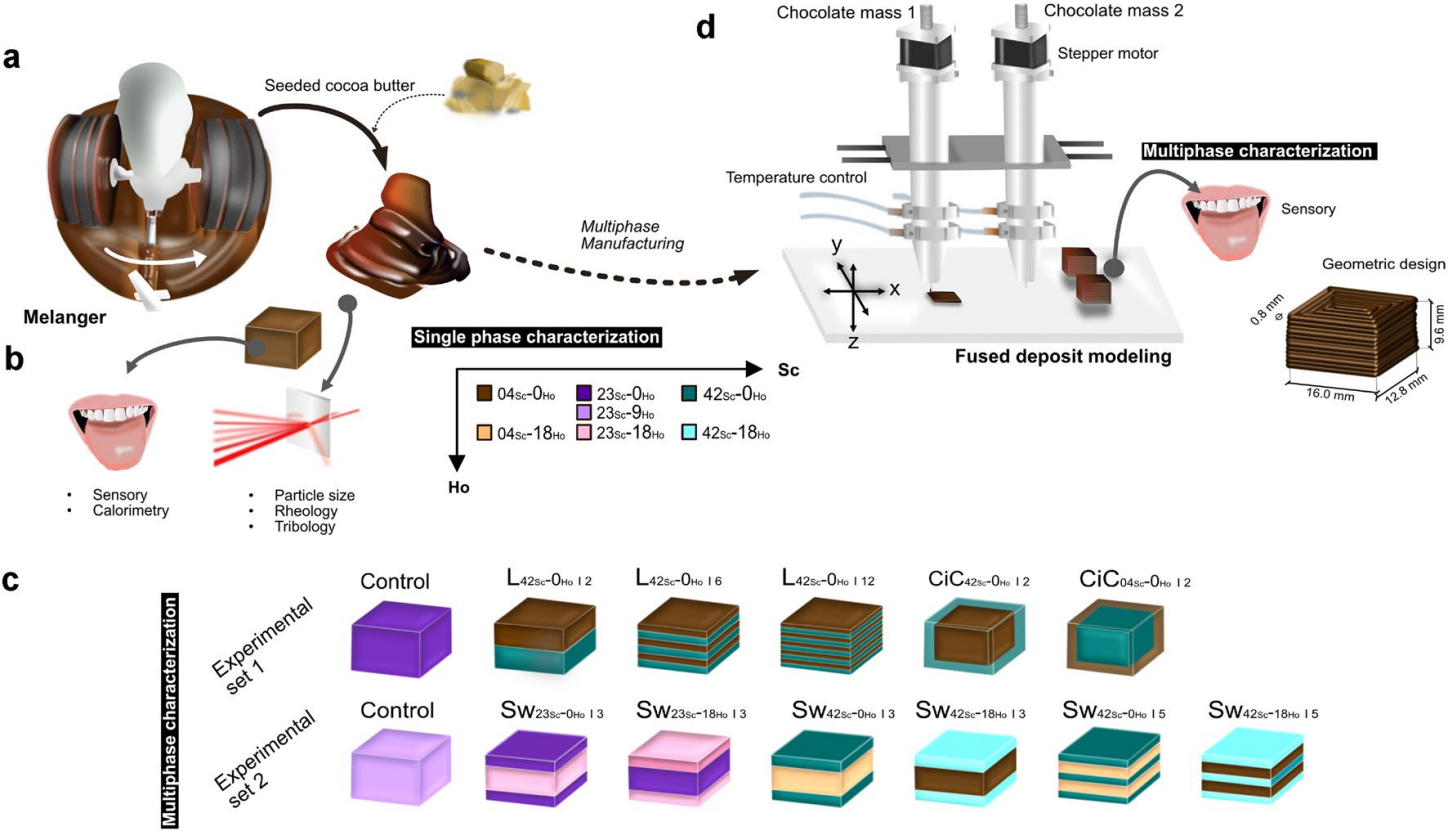
(**a**)–(**c**). A detailed overview of the preparation of the chocolate masses and the additively manufactured multiphase structures can be found in section (**d**). Multiphase structures were subjected to two different sensory sessions, referred to as experimental sets 1 and  2. All structures were prepared using one (for control) or two sensorially characterized chocolate masses. In addition, the chocolate masses were analytically evaluated by calorimetry, rheology, tribology and laser diffraction, as described in section (**b**). In the first set of experiments, sugar was inhomogeneously distributed within *cube-in-cube* (*CiC*) and *layered* (*L*) structures, varying the manufacturing effort and complexity of the latter by changing the number of layers. The second set of experiments focused on *sandwich* designs, where the number of layers and the composition of each layer in terms of sugar (*Sc*) and hazelnut oil (*Ho*) content were varied. Importantly, the total content of sugar and hazelnut oil was maintained constant across these designs.

All chocolate samples listed in Table 1 were standardized with respect to solids content (%w/w), and total fat content (%w/w). The sugar content in the samples ranged from 4 %w/w to 42 %w/w. This was achieved by replacing sugar (sourced from Zuckerfabriken Aarberg AG, Switzerland) with varying amounts of polydextrose (Litesse®Two Powder (IP Grade), Danisco AG, Netherlands) and inulin (OraftI®HSI, BENEO-Orafti S.A., Belgium) in a constant ratio of 2.17:1 (polydextrose to inulin). All non-cocoa ingredients, i.e.  polydextrose, inulin, and sucrose, were pre-mixed with melted cocoa mass (“Rondo”, Max Felchlin AG, Switzerland) in a blender (Titanium KMT056, Kenwood Swiss AG, Switzerland) before being transferred to a small melanger (ECGC-12SLTA, Cocoatown LLC, USA). Additional melted cocoa butter or hazelnut oil (Haselnussöl, Demeter, Switzerland), the latter added in the range of 0 to 18 %w/total fat, was added to the mixture after 90 minutes of refining. A detailed description of the preparation and the subsequent molding process (for single mass sensory and instrumental analysis) can be found in Burkard et al.^23^. For molding, the seeded chocolate masses were transferred into silicone molds (Silicone Culinair®, Siliconesandmore, Netherlands) of defined geometric design (16 mm x 9.6 mm x 12.8 mm). A schematic overview of the mass production can be found in Fig. 5a and the following analytical and sensory characterizations are shown in Fig. 5b.

**Table 1:** Composition of chocolate masses *04*$_{Sc}$-*0*$_{Ho}$, *04*$_{Sc}$-*18*$_{Ho}$, *23*$_{Sc}-$*0*$_{Ho}$, *23*$_{Sc}$-*9*$_{Ho}$, *23*$_{Sc}$-*18*$_{Ho}$, *42*$_{Sc}$-*0*$_{Ho}$, *42*$_{Sc}$-*18*$_{Ho}$. The chocolate masses were labeled using the syntax *Sugar (%w/w)*$_{Sc}$-*Hazelnut oil (%w/w)*$_{Ho}$ content, where *Sc* represents the sucrose content of the chocolate masses, while *Ho* denotes the proportion of hazelnut oil relative to the total fat content of the samples.

Composition	Chocolate masses						
	${04}_{{Sc}}$-${0}_{{Ho}}$	${04}_{{Sc}}$-${18}_{{Ho}}$	${23}_{{Sc}}$-${0}_{{Ho}}$	${ 23}_{{Sc}}$-${9}_{{Ho}}$	${23}_{{Sc}}$-${18}_{{Ho}}$	${42}_{{Sc}}$-${0}_{{Ho}}$	${42}_{{Sc}}$-${18}_{{Ho}}$
Sugar content (%w/w)	04.15	04.15	23.00	23.00	23.00	41.71	41.71
Hazelnut content/ total fat (%w/w)	00.00	17.96	00.00	08.98	17.96	00.00	17.96
Total fat (%w/w)	35.09	35.09	35.09	35.09	35.09	35.09	35.09
Cocoa mass (%w/w)	50.70	50.70	50.70	50.70	50.70	50.70	50.70
Sugar (%w/w)	04.15	04.15	23.00	23.00	23.00	41.71	41.71
Inulin (%w/w)	11.83	11.83	05.55	05.55	05.55	00.00	00.00
Polydextrose (%w/w)	25.73	25.73	13.16	13.16	13.16	00.00	00.00
Cocoa butter (melted) (%w/w)	06.30	00.00	06.30	03.15	00.00	06.30	00.00
Cocoa butter (crystallized) (%w/w)	00.90	00.90	00.90	00.90	00.90	00.90	00.90
Hazelnut oil (%w/w)	00.00	06.30	00.00	03.15	06.30	00.00	06.30
Soy lecithin (%w/w)	00.39	00.39	00.39	00.39	00.39	00.39	00.39

### Single mass characterization

#### Particle size analysis

The particle size distribution of the chocolate masses was measured using a Beckman laser diffractometer (13 320 XR, Beckman Coulter Inc. , United States). For sample preparation, the melted chocolate masses were suspended in Hydriol®SOD.24 (Hydrior AG, Wettingen, Switzerland) and the instrument was operated with Hydriol®SOD.24 as solvent. Prior to measurement, the suspended sample was sonicated for two minutes at 65 Hz in an ultrasonic bath (Elmasonic P 30 Hat, EMAG Nänikon, Switzerland) at $50\,^{\circ }\hbox {C}$. The diffraction pattern was interpreted using the Fraunhofer model^60^. All samples were measured in triplicate (N = 3).

#### Rheology

Rheological properties were measured using a rheometer (MCR 302, Anton Paar, Austria). Approximately 35 g of chocolate was melted at $50\,^{\circ }\hbox {C}$, thoroughly mixed with 0.36 g of crystallized cocoa butter, and transferred to a couette flow cell (CC27, Anton Paar, Austria) at $40\,^{\circ }\hbox {C}$. The sample was presheared for two minutes at a shear rate of 10 s$^{-1}$ at $40\,^{\circ }\hbox {C}$. For a logarithmic shear rate ramp ranging from 0.1 s$^{-1}$ to 100 s$^{-1}$, ten data points per decade were collected. All data points were collected under steady state conditions. All measurements were performed in triplicate (N = 3) and analyzed in Origin (OriginPro 2021, 9.8.0.200).

#### Differential scanning calorimetry

The melting profiles of the chocolate masses were characterized by differential scanning calorimetry (DSC 3+/500, Mettler Toledo GmbH, Switzerland). An indium and a water sample were used for calibration. 5 ± 0.2 mg of tempered chocolate was weighed into a $30\,\upmu \hbox {L}$ aluminum crucible (Mettler Toledo GmbH, Switzerland). For measurement, the sample was equilibrated at $20\,^{\circ }\hbox {C}$ for two minutes, followed by a linear heating ramp to $50\,^{\circ }\hbox {C}$ at a heating rate of 4 K/min. All samples were measured in triplicate (N = 3). Curve characteristics such as onset, offset, peak melting temperatures and melting enthalpy were extracted using Origin (OriginPro 2021, 9.8.0.200) for analysis.

### Multiphase structure manufacturing

The multiphase structures were labeled with respect to the experimental design, referring to the chocolate mass initially placed on the tongue, and the number of layers (*design*$_{chocolate mass\,on\,tongue\,\vert \,number\,of\,layers}$). The design notation consisted of abbreviations for *layered* (abbr.  *L*) or *cube-in-cube* (abbr.  *CiC*) designs, followed by the specific chocolate mass and the number of layers (e.g., *Sw*${_{23}}{_{Sc}}$-*0*$_{Ho \,\vert \,3}$ was a 3-layer *sandwich* structure with a bottom and top layer composed of chocolate with 23 %w/w sugar content without hazelnut oil).

In the first set of experiments, three *layered* structures, two *cube-in-cube* structures, and a homogeneous control with no sugar gradient and a sugar content of 23 %w/w, were prepared. All multiphase structures in the first set of experiments were made from chocolate with high (*42*$_{Sc}$-*0*$_{Ho}$) and low (*04*$_{Sc}$-*0*$_{Ho}$) sugar content. The number of layers of the *layered* structures varied, allowing to account for structural complexity: either two (*L*${_{42}}{_{Sc}}$-*0*$_{Ho\,\vert \,2}$), six (*L*${_{42}}{_{Sc}}$-*0*$_{Ho\,\vert \,6}$), or twelve (*L*${_{42}}{_{Sc}}$-*0*$_{Ho\,\vert \,12}$), whereby the sweet layer was always placed in contact with the participants’ tongues. For the *cube-in-cube* structures, high-sugar (*CiC*${_{42}}{_{Sc}}$-*0*$_{Ho \,\vert \,2}$) or low-sugar chocolate (*CiC*${_{04}}{_{Sc}}$-*0*$_{Ho \,\vert \,2}$) completely enveloped the other chocolate mass.

In the second set of experiments, *sandwich* structures were designed, in which the top and bottom layers were composed of the same masses, a distinct difference from the *layered* configurations. In addition, a fraction of the cocoa butter was replaced with hazelnut oil (*Ho*). As a result, six *sandwich* designs were prepared that differed in the number of layers (either three or five) and the composition of the top/bottom chocolate mass, resulting in the multiphase structures *Sw*${_{42}}{_{Sc}}$-*18*$_{Ho\,\vert \,5}$, *Sw*${_{42}}{_{Sc}}$-*18*$_{Ho\,\vert \,3}$, *Sw*${_{42}}{_{Sc}}$-*0*$_{Ho\,\vert \,3}$, *Sw*${_{42}}{_{Sc}}$-*0*$_{Ho\,\vert \,5}$, *Sw*${_{23}}{_{Sc}}$-*0*$_{Ho\,\vert \,3}$, and *Sw*${_{23}}_{{Sc}}$-*18*$_{Ho \,\vert \,3}$. Analogous to the first set of experiments, all multiphase structures were evaluated against a homogeneous control that maintained the same sugar content and, due to the introduction of hazelnut oil, the same hazelnut oil content (9 %w/total fat).

The dimensions of the multiphase structures were set at 16 mm x 9.6 mm x 12.8 mm. The total sugar (23 %w/w) and hazelnut oil (0 %w/total fat for the first, and 9 %w/total fat for the second experiment) contents were held constant with respect to the homogeneous control (notated as *23*$_{Sc}$-*0*$_{Ho}$ for experimental set 1 and *23*$_{Sc}$-*9*$_{Ho}$ for experimental set 2). A comprehensive illustration of the experimental setup and the multiphase structures is provided in Fig. 5d.

All chocolate masses were seeded with 1 %w/w of $\beta _{V/VI}$ cocoa butter prior to being transferred to stainless steel cartridges, equipped with a 15 mm long nozzle with an inner diameter of 0.8 mm (Sigrist & Partner AG, Matzingen, Switzerland). To maintain a constant product temperature, both the cartridge and nozzle temperature were set at 32.5 ± 0.3 $^{\circ }\hbox {C}$ using an aluminum jacket connected to a tempered water bath (Julabo 9153618 FP35, JULABO GmbH, Germany). FDM printing was performed using a custom-built three-axis Cartesian printer, designed by the Institute for Print Technology (Bern University of Applied Sciences)<f. The printer setup is shown in Fig. 5c. It was placed in an environmental chamber (KK-1000 CHLT, Kambic, Slovenia) to maintain the ambient temperature at $10\,^{\circ }\hbox {C}$. Printer settings, including print speed, were defined in the Slic3r software (version 1.3.1). Printer operation was controlled by the Repetier Host software (version 2.1.6, 2019). After printing, all samples were stored at $5\,^{\circ }\hbox {C}$ prior to sensory testing. All printer settings are detailed in Supplementary Information 1.

### Sensory analysis

All participants (ten women aged between 35 and 55 years) were recruited by the HAFL Sensory Evaluation Department and participated in 21 sensory sessions. In accordance with the constraints imposed by the COVID-19 pandemic, experimental set 1 was conducted at home, while all other tests were conducted in the HAFL sensory laboratory. The sensory analysis consisted of an in-depth study of the individual chocolate masses, and of a sensory characterization of the 3D-printed multiphase structures during two experimental sets. All sensory experiments were performed by the same group of participants (N = 8), while two participants were replaced during experimental set 2.

A comprehensive summary of all sensory experiments is presented in Table 2. All test sessions/methods were conducted in accordance with the relevant ISO standards and guidelines (ISO 6658 217 (E)), were approved by the University (BFH-HAFL) and guided by a sensory expert from HAFL. To ensure standardized consumption, participants were instructed to place the sample in their mouth and press it lightly against their palate. They were allowed to swallow if necessary and to move their tongue periodically during consumption. Consumption was considered complete once the entire sample had been swallowed. Still water (at $25\,^{\circ }\hbox {C}$) and unsalted crackers (M-Classic Microc, Migros SA, Switzerland) were provided to clean the palate between samples. Prior to enrollment, all participants signed an informed consent document indicating their understanding and acceptance of the study procedures. To assess the panel’s effectiveness, we first evaluated individual performance in terms of discrimination, agreement, and repeatability. The panel’s collective performance was then evaluated using the same criteria and can be found in Supplementary Information 11.

**Table 2:** Overview of all sensory tests conducted, both on single-mass and on multiphase structures: methodology used, time sequence of the analysis, experiment details, and attributes investigated. The sensory tests were always preceded by training sessions.

Analyzed sample	Methodology	Sequence	Training	Evaluations	Remarks	Attributes
Single mass	CATA	1	0	1	-	Flavor (texture, taste and aroma)
	Comparative profiling	2	3	2	-	Sweetness, smoothness, bitterness (maximum) and time of melting
Multiphase structures	Progressive profiling	3	4	2	Experiment 1	Sweetness (start, max and end)
	Comparative profiling	4	2	2	Experiment 2 –	Sweetness, smoothness, bitterness (maximum and overall) and time of melting
	TI	5	3	2	Experiment 2 –	Sweetness (temporal)

#### Single mass characterization

**Check-all-that-apply:** First, participants performed a check-all-that-apply (CATA) test to describe the sensory profiles of four defined chocolate masses (*04*$_{Sc}$-*0*$_{Ho}$, *04*$_{Sc}$-*18*$_{Ho}$, *42*$_{Sc}$-*0*$_{Ho}$ and *42*$_{Sc}$-*18*$_{Ho}$). These masses were chosen with the expectation that they would cover the full range of sensory perception. Panelists were presented with a list of ten predefined attributes (see Supplementary Information 2), and asked to select the attributes that best described each sample.

**Comparative profiling:** From the CATA results detailed in Supplementary Information 6, the attributes bitterness, smoothness, and total consumption time were identified as significant in differentiating the chocolate masses. Over three sessions, participants were trained to ensure consistent ratings for the three attributes in addition to sweetness. They practiced rating the maximum intensity of each attribute on a sensation scale ranging from 0 (not bitter/sweet/smooth) to 100 (extremely bitter/sweet/smooth). Participants consumed the control sample *23*$_{Sc}$-*9*$_{Ho}$ and were instructed to rate its maximum sensation as 50 for all attributes. The rating of sweetness and smoothness was practiced with defined test samples (see Supplementary Information 3). The samples were tempered to 20 ± 1 $^{\circ }\hbox {C}$ and served on plastic trays with random three-digit codes. Each session was conducted by a sensory panel leader, a control warm-up sample was presented at the beginning of each session and the samples were presented in a balanced order. A blind control was also included in the experimental design. Data were collected using EyeQuestion (licensed to HAFL, Zollikofen, Switzerland), including melting time, defined as the time between the start of oral processing and the moment when the chocolate had been completely swallowed.

#### Multiphase structure analysis

**Progressive profiling:** Multiphase structures in the first experimental set ( *L*${_{42}}{_{Sc}}$-*0*$_{Ho\,\vert \,2}$, *L*${_{42}}{_{Sc}}$-*0*$_{Ho\,\vert \,6}$, *L*${_{42}}{_{Sc}}$-*0*$_{Ho\,\vert \,12}$, *CiC*${_{42}}{_{Sc}}$-*0*$_{Ho\,\vert \,2}$ and *CiC*${_{04}}{_{Sc}}$-*0*$_{Ho\,\vert \,2}$), and a homogeneous control (*23*$_{Sc}$-*0*$_{Ho}$) were evaluated at three time points t$_{start}$, t$_{max}$, and t$_{end}$. Similar to the chocolate masses comparative profiling method, participants were asked to rate the sweetness sensation on a scale from 0 (not sweet) to 100 (extremely sweet). Each session began with a warm-up sample of a defined maximum sweetness intensity of 50. In addition to rating the maximum sweetness intensity I$_{max}$, participants were instructed to rate the initial (I$_{start}$) and final sweetness perception (I$_{end}$), the latter being defined as the sensation before the last piece of sample was completely swallowed. Due to the pandemic, the training and testing sessions were carried out online: the participants were at home and guided remotely by a sensory panel leader. Data from both sessions were recorded using EyeQuestion (licensed to HAFL, Zollikofen, Switzerland).

**Comparative profiling:** All multiphase structures in the second experimental set (*Sw*${_{42}}{_{Sc}}$-*18*$_{Ho\,\vert \,3}$, *Sw*${_{42}}{_{Sc}}$-*18*$_{Ho\,\vert \,5}$, *Sw*${_{42}}{_{Sc}}$-*0*$_{Ho\,\vert \,3}$, *Sw*${_{42}}{_{Sc}}$-*0*$_{Ho\,\vert \,5}$, *Sw*${_{23}}{_{Sc}}$-*0*$_{Ho\,\vert \,3}$, *Sw*${_{23}}{_{Sc}}$-*18*$_{Ho\,\vert \,3}$) were evaluated by comparative profiling on a unipolar scale from 0 to 100. In addition to rating the maximum perception of the attributes sweetness, smoothness, and bitterness, participants also rated the overall perception of these attributes. After three training sessions, the panel rated the samples twice in two test sessions.

**Time-intensity:** During three training sessions, participants were instructed to rate the temporal intensity of the sweetness. Specifically, the participants were instructed to begin rating the sweetness intensity after 10 seconds of consumption and continue at 0.1 Hz intervals for a total of three ratings. After this initial period, participants were allowed to continue rating the samples at their own pace. To provide further guidance, participants were asked to rate the intensity when the sample had completely melted (I$_{melt}$), as well as the final perceived sweetness sensation (I$_{end}$) upon swallowing. Further details can be found in Supplementary Information 4.

### Descriptive statistics

A comprehensive breakdown of the statistical analyses is provided in Supplementary Information 5. The results from the Check-All-That-Apply (CATA) approach were analyzed using Cochran’s Q tests to identify significant differences among the attributes. For all other sensory evaluation data, we employed linear mixed-effects models in RStudio to account for the fixed and random effects within our experimental design. Significant findings (*p* < 0.05) were further explored with a Tukey Honestly Significant Difference (HSD) post-hoc test to pinpoint specific group differences. For an in-depth review, of the specific models applied to each sensory evaluation, please refer to the details in Supplementary Information 5.

## Supplementary Information

Supplementary Information.

## Data Availability

The datasets generated during and/or analyzed during the current study are available from the corresponding author upon reasonable request. The Python script for calculating the time-intensity curves can be found at https://github.com/burkardj/TI-Processing/.
