# Supplementary material for: Exploring the effects of structure and melting on sweetness in additively manufactured chocolate
Source: Sci Rep. 2024 Apr 9;14:8261. doi: 10.1038/s41598-024-58838-6 (PMC11372135; doi:10.1038/s41598-024-58838-6)
Supplement: Supplementary file 1 — Supplementary Information. [file 41598_2024_58838_MOESM1_ESM.pdf]

Supplementary Information

Johannes Burkard[1,2,*], Lucas Kohler[1], Sophia Caciagli[1], Nicolas Herren[1], Mark Kozamernik[2], Saskia Mantovani[2], Erich J Windhab[1], and Christoph Denkel[2,*]

[1]ETH Zurich, Institute of Food, Nutrition and Health, 8092 Zürich, Switzerland
[2]Bern University of Applied Sciences, School of Agricultural, Forest and Food Sciences HAFL, Food Science and Management, 3052 Zollikofen, Switzerland
[*]johannes.burkard@hest.ethz.ch
[*]christoph.denkel@bfh.ch

1 Printer settings

Printer and firmware settings used for FDM printing.

Supplementary Table S 1. Printer Firmware Settings (EEPROM).

Firmware Settings	*Extruder 1*	*Extruder 2*
X-Axis (steps/mm)	266	266
Y-Axis (steps/mm)	266	266
Z-Axis (steps/mm)	1000	1000
X-Axis acceleration (mm/s^2)	1000	1000
Y-Axis acceleration (mm/s^2)	1000	1000
Z.Axis acceleration (mm/s^2)	100	100
Extr X-Offset (steps)	0	32000
Extr Y-Offset (steps)	0	200

Supplementary Table S 2. Printer Settings.

Printer Settings	*Extruder 1/2*
Printer speed (mm/s)	15
Nozzle diameter (mm)	0.8
Layer height (mm)	0.8
First layer height (mm)	0.8
Vertical shell perimeter (-)	1
Horizontal shell top (-)	1
Horizontal shell bottom (-)	1
Seam position	Nearest
Fill density (%)	100
Fill pattern	Concentric
Top/Bottom fill pattern	Rectilinear
Skirt	Each phase change
Skirt loops (-)	2
Skirt distance from object (mm)	10

2 Attribute list for CATA

Participants conducted a check-all-that-apply (CATA) test to describe the sensory profiles of four selected chocolate masses (04_{Sc}-0_{Ho}, 04_{Sc}-18_{Ho},42_{Sc}-0_{Ho} and 42_{Sc}-18_{Ho}). They were presented with a list of ten pre-defined attributes (see Table S 3 to select attributes for each sample).

Supplementary Table S 3. Definition of sensory attributes

Attribute	Definition
Sweetness	Intensity of the basic sweet taste (e.g., sucrose in water)
Bitterness	Intensity of the basic bitter taste (e.g., caffeine in water)
Acidity	Intensity of the basic acid taste (e.g., citric acid in water)
Cocoa	Intensity of the generic cocoa flavour (100% cocoa powder)
Fatty	Intensity of the fat taste (e.g., butter)
Melting	Ability of the product to become liquid or semi-liquid
Smoothness	Absence of a sandy feeling in the mouth
Stickiness	Ability of the product to stick between the tongue and the palate
Mouth coating	Ability of the product to cover the palate and tongue with a layer
Astringency	Intensity of the drying sensation in the mouth (e.g., red wine)

3 Attribute calibration for sensory testing

Prior to evaluating bitterness, sweetness, and smoothness, a series of anchor points were established to calibrate the smoothness and sweetness scales, with values ranging from 0 to 100. The reference probe, designated as 23_{Sc}-09_{Ho}, was assigned a midpoint value of 50 on each scale (bitterness, sweetness, and smoothness). As part of this calibration process, sucrose was reconstituted in tap water at the concentrations listed in the Supplementary Table S 4. The relationship between sucrose concentration and sweetness intensity, as previously described by Karalus *et al.*[1], was used. In addition, anchor points for smoothness were calibrated by adjusting the refining time from 1.5 to 12 h and by including chocolate masses with higher hazelnut oil content at higher smoothness ratings (see Supplementary Table S 5). Calibration for both smoothness and sweetness was practiced during panel training for single and multiphase structures.

Supplementary Table S 4. Calibration standards for sweetness intensity during comparative profile testing.

Terminology	*Sucrose concentration (g/L)*	*Sweetness rating (-)*
Much lower	30	20
Slightly lower	40	35
Reference	50	50
Slightly higher	60	65
Much higher	70	80

Supplementary Table S 5. Calibration standards for smoothness intensity during comparative profile testing.

Chocolate Masses	*Refining time (h)*	*Smoothness rating (-)*
23_{Sc}-0_{Ho}	1.5	10
23_{Sc}-0_{Ho}	7	40
23_{Sc}-9_{Ho}	7	50
23_{Sc}-18_{Ho}	7	60
23_{Sc}-18_{Ho}	12	75

4 Time-intensity methodology

Time-intensity (TI) measurements were performed to measure the temporal sweetness perception. Panelists rated a total of seven samples, including a homogeneous control and six multiphase structures. The tasting protocol was similar to the comparative profiling protocol, and panelists were instructed to rate sweetness intensity every ten seconds for the first three ratings (10, 20, and 30 s). Beyond that point, panelists could choose their rating frequency. However, two additional fixed rating points were established: I_{melt}, the point at which the sample was completely melted, and I_{end}, the rating just before swallowing (Supplementary Fig. S 1).

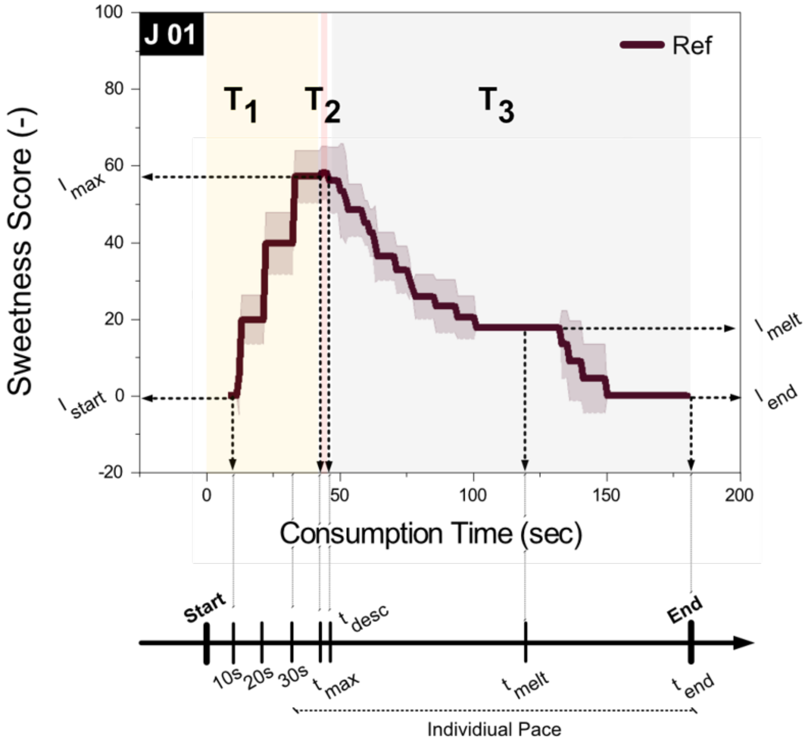

Supplementary Figure S 1. The intensity sampling process is illustrated at various time points, as indicated on the time bar beneath the exemplary time-intensity (TI) curve. This curve represents the average and normalized response for the control sample (Ref) as perceived by panelist 1 (J01). The initial three intensity ratings are captured at intervals of 10, 20, and 30 seconds, followed by ratings at a pace determined individually by the panelist. Two additional fixed rating points, I_{melt} and I_{end}, are also recorded. I_{melt} corresponds to the moment when the sample has completely melted, and I_{end} is noted just before swallowing the sample. The TI curve was normalized separately for each of the three time domains (T_1, T_2, and T_3).

The method was practiced in three sessions and evaluated in two separate sessions. For all sessions, the multiphase structures in experimental set 2 were rated sensorially. The data obtained were averaged based on the normalization of individual curves for both intensity and time. According to the Supplementary equation (1), the individual dynamic intensity correction I'(t) was based on the normalization of the individual intensity I(t) with the ratio of the overall maximum intensity I_{max,P_j} to $I_{max,i}$.

$$I(t)'_{P_j} = \frac{I_{max,P_j}}{I_{max,i}} * I(t) \tag{1}$$

To accommodate x-axis normalization for non-monotonic curve shapes, simple averaging over intensity can lead to a loss of information about individual trends, especially from outliers or a minority pattern[2,3]. Our method is based on Liu *et al.*[3]. We are referring to the following parameters, describing all parameters relevant for TI measurements:

- Maximum intensity (I_{max}): largest registered value for perceived intensity.
- Starting time (t_{start}): first value of time when intensity is greater than 0.
- Maximum time (t_{max}): the first time at which maximum intensity registered.

- Decline time ($t_{decline}$): the time at which the curve starts to decline from maximum intensity and the last time at which a maximum intensity is registered.
- Finish time (t_{end}): the time when the intensity first returns to 0, which is also called persistence time, or extinction time.
- Recording time: from time set to 0 to end of recording.

In addition to I_{max}, four "time landmarks" were averaged, namely starting time, time to maximum, time at which the curve begins to descend from I_{max} and ending time. The time normalization takes three time domains (T_1, T_2, T_3) into account, framed by the landmarks introduced above (Supplementary Fig. S 1). The curves were then normalized in the t-direction to have the same t_{start}, t_{max}, $t_{decline}$, and t_{end} per product, according to Supplementary equation (2).

If $t_{decline}$ was equal to t_{max}, t' per product was reduced to the normalization of t' with the intervals (1) and (3). If I_{end} was not zero, the curve was discarded. To average the values of I, each curve per product cluster was divided into n equal time intervals (n = 60) and arithmetically averaged as a master curve per product. The automatic averaging method was performed with a Python script, loading the raw text files. The Python script is uploaded at https://github.com/burkardj/TI-Processing/ . An overview of all normalized and averaged TI curves, separated by panelist, is shown in Supplementary Fig. S 2.

$$t' = \begin{cases} \mathbf{T_1:}\ \left(\frac{t_{max}-t_{start}}{t_{max,i}-t_{start,i}}\right)(t-t_{start,i})+t_{start}\ when\ t_{start,i} \leq t \leq t_{max,i}\ (1) \\ \mathbf{T_2:}\ \left(\frac{t_{dec}-t_{max}}{t_{dec,i}-t_{max,i}}\right)(t-t_{max,i})+t_{max}\ when\ t_{max,i} < t < t_{dec,i}\ (2) \\ \mathbf{T_3:}\ \left(\frac{t_{end}-t_{dec}}{t_{end,i}-t_{dec,i}}\right)(t-t_{dec,i})+t_{dec}\ when\ t_{dec,i} \leq t \leq t_{end,i}\ (3) \end{cases} \quad (2)$$

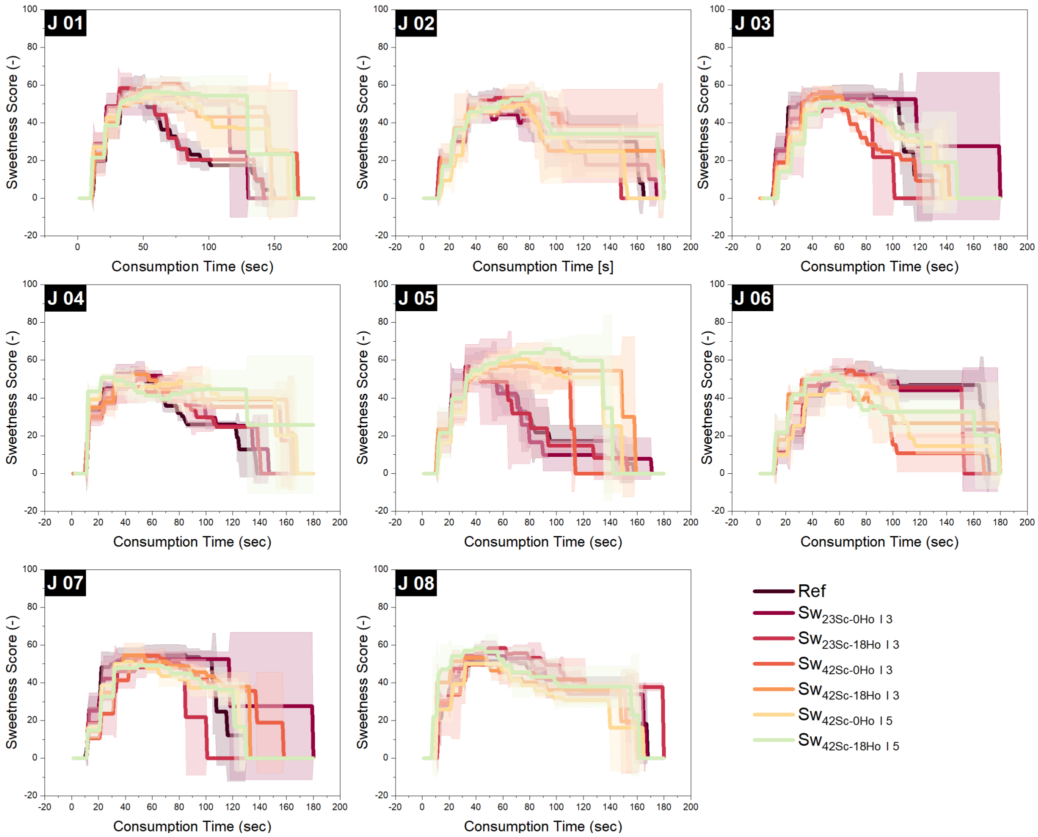

Supplementary Figure S 2. Normalized TI data, separated by panelists (J01-08).

5 Descriptive Statistics

Single mass sensory and instrumental analysis: The significance of the CATA data was determined using Cochran's Q test (coins library[4]) in RStudio (2023.03.0 Build 386, Posit Software, PBC). A multivariate comparison was performed in RStudio to compare the ratings of the attributes sweetness, smoothness, and bitterness among the individual masses. To better distinguish the differences between the products and the homogeneous control, individual intensity ratings were obtained by subtracting the control rating, with both ratings given by the same participant within a single session. These adjusted differences were then averaged to calculate the average relative difference from control (ARD) for each product. The ARD was defined as follows:

$$ARD_p\,(\%) = \frac{1}{j*i}\sum_{i}^{2}\sum_{j}^{8}\frac{y_{ijp}-c_{ij}}{c_{ij}}*100 \quad (3)$$

where p is the number of products, i is the number of sessions, j is the number of panelists, y is the rating for a given product, session, and panelist, and c is the control rating for a given session and panelist.

$$\text{Sensory rating}(y) \sim \underbrace{Sc*Ho}_{\text{Product}} + (Sess:Seq) + (1|Pan) \quad (4)$$

Sensory ratings (y) (smoothness, sweetness, and bitterness) served as the dependent variables in the linear mixed-effects model, as shown in Supplementary equation (4) (lme4 library, lmer test library[5]). Each sample was uniquely identified by its sample code (e.g., 23_{Sc}-9_{Ho}) or by its hazelnut oil content (*Ho*) and its sugar content (*Sc*). These identifiers, along with the interaction between the sessions and the sequence order (*Sess : Seq*), were treated as fixed effects. The model also included an intercept and a random error, to account for participant variability *(1 | Pan)*. Model diagnostics were checked using the DHARMa package[6]. For significant results ($p < 0.05$), a pairwise comparison was performed using a Tukey HSD post-hoc test that controls the family-wise error rate (emmeans library[7]).

$$\text{Mass characteristics}(y) \sim \underbrace{Sc*Ho}_{\text{Product}} + (1|Rep) \quad (5)$$

As with the sensory data, the instrumental data (calorimetry, rheology and particle size) were analyzed using a linear mixed-effects model, as shown in Supplementary equation (5). The data were grouped into two batches (= sessions), with three replicates per batch. Various measurements, such as d_{90}, were used as dependent variables (y). Meanwhile, hazelnut oil (*Ho*), sugar content (*Sc*), or sample ID (*Product*), were set as fixed factors. An intercept was included with the batches serving as a random error *(1 | Rep)*. Similarly to the sensory analysis, the normality of the models was checked with the DHARMa package[6] and significant results were analyzed with a post-hoc Tukey test (Agricolae library[8]).

To investigate correlations between sensory and instrumental variables, a multi-comparison non-parametric Spearman correlation plot was designed (library ggstatsplot[9]). Significant pairwise interactions were detected with an unpaired Wilcoxon t-test (library ggstatsplot[9]).

Multiphase structures | progressive profiling: During progressive profiling, the ARD for sweetness intensity at I_{start}, I_{max}, and I_{end} were defined as the dependent variables (y) in Supplementary equation (6). Session and order were treated as fixed effects (*Sess : Seq*), product was further distinguished by sugar content *Sc*, structure (*Struct*, either *cube-in-cube* or *layered*) and number of layers (*Lay*). Participant *(1 | Pan)* was set as a random error. Significant results ($p < 0.05$) were compared using a Tukey post-hoc test (Agricolae library[8]), and model metrics were checked using the DHARMa package[6].

$$\text{Sweetness intensity}(y) \sim \underbrace{Sc+Struct+Lay}_{\text{Product}} + (Sess:Seq) + (1|Pan) \quad (6)$$

Multiphase structures | comparative profiling: As for the single mass profiling, the statistical analysis was performed with multivariate comparison (see Supplementary equation (7)). The ARD of the attributes smoothness, sweetness, and bitterness (maximum and total) were set as dependent variables (y), while sugar (Sc) content, hazelnut oil (Ho) content and the number of layers (Lay) were treated as fixed factors. Session and order were treated as linear fixed effects ($Sess : Seq$). Participant *(1 | Pan)* was set as a random error. Significant results ($p < 0.05$) were compared using a Tukey post-hoc test (Agricolae library[8]). Model normality was checked using the DHARMa package[6].

$$\text{Sensory rating}(y) \sim \underbrace{Sc + Ho + Lay}_{\text{Product}} + (Sess : Seq) + (1|Pan) \tag{7}$$

Multiphase structures | time-intensity: The time-intensity (TI) data were averaged across all participants for the two test sessions, based on normalization of individual curves for both intensity and time. The time-intensity correction is based on Liu *et al.*[3]. A detailed normalization and averaging procedure can be found in Supplementary Information 4. A number of key values (e.g., time to peak or baseline intensity) were further analyzed, based on Supplementary equation (8), with the key values or product (*TI-value*) set as the dependent variable. Samples and order were treated as fixed factors, while panelists were set as random errors. Tukey was used as a post-hoc test to further compare the means of the key values (Agricolae library[8]). The normalization and averaging method was performed with a Python script (Jupyter pyi version 3.6.3).

$$\text{TI-value}(y) \sim Value + (Sess : Seq) + (1|Pan) \tag{8}$$

6 CATA results

CATA results summarized in Supplementary Fig. S3 and Supplementary Table S 6.

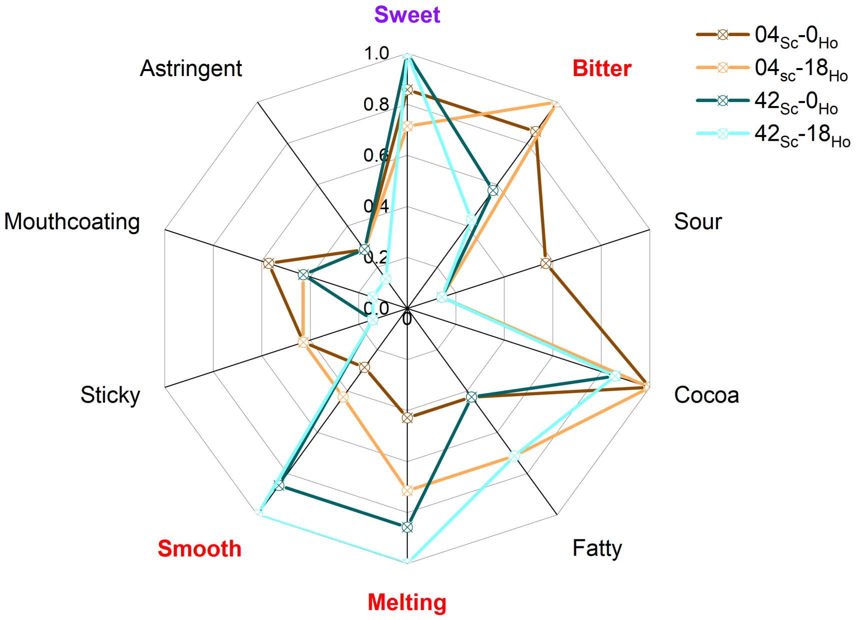

Supplementary Figure S 3. Check-all-that-apply data generated from the evaluation of the four chocolate masses identified as 04_{Sc}-0_{Ho}, 04_{Sc}-18_{Ho}, 42_{Sc}-0_{Ho}, and 42_{Sc}-18_{Ho}. Attributes highlighted in red were significantly different and were selected for further sensory analysis in addition to the sweetness attribute highlighted in purple.

Supplementary Table S 6. Cochran's Q analysis was performed on sensory attributes identified during the CATA of the following products: 04_{Sc}-0_{Ho}, 04_{Sc}-18_{Ho}, 42_{Sc}-0_{Ho}, and 42_{Sc}-18_{Ho}. P-values below a level of 0.05 indicate significance. The test statistic Q and the degree of freedom df per attribute are also listed.

	Q	df	p-value
Sweet	4.71	3	0.194
Bitter	8.57	3	0.036
Sour	2.67	3	0.446
Cocoa	3	3	0.392
Fatty	2.4	3	0.494
Melting	8.08	3	0.044
Smoothness	11.33	3	0.01
Sticky	1.74	3	0.629
Astringent	7.71	3	0.052
Mouth Coating	3	3	0.392

7 Pairwise comparison of analytical attributes

Supplementary Table S 7 summarizes the means for each instrumental measure used during the analysis for all chocolate masses.

Supplementary Table S 7. Summary of the attributes of all chocolate masses (particle size, rheology and calorimetry), presented with their respective mean and standard deviation. Capital letter (A to G) identify the chocolate masses. The letters are used to denote significant differences between chocolate masses. For example, if the chocolate mass 04_{Sc}-18_{Ho} (B) differs significantly from a reference chocolate mass (e.g., chocolate mass A) for a particular attribute, a letter is added as a superscript next to the number. This letter indicates a significant difference from the reference mass A, with 0,1 or 2 apostrophes (') indicating the level of significance (A for $p < 0.05$, A' for $p < 0.01$, A" for $p < 0.001$).

**Chocolate Mass (Letter)**	**Particle Size Distribution**			**Rheology**		**Differential Scanning Calorimetry**			
	Span (-)	d_{90} (µm)	$\eta_{10}(Pa*s)$	$\eta_{20}(Pa*s)$	$\eta_{10}(Pa*s)$	Enthalpy (J/g)	Onset (°C)	Peak (°C)	Offset (°C)
04_{Sc}-0_{Ho} (A)	3.1	29.3 ± 0.5	6.4 ± 0.4^{F}	5.2 ± 0.3	4.4 ± 0.3	-44.8 ± 1.7	26.3 ± 0.3	32.8 ± 0.5	34.6 ± 0.4
04_{Sc}-18_{Ho} (B)	2.9 ± 0.2	26.5 ± 3.2	5.6 ± 0.1	4.9	4.2	$-37 \pm 0.5^{A".C",D",F"}$	$25.3 \pm 0.1^{B",D}$	32.1 ± 0.4	$33.8 \pm 0.2^{A",C,F}$
23_{Sc}-0_{Ho} (C)	3.1 ± 0.2	26.0 ± 2.0	6.4 ± 0.1	5.0	4.2 ± 0.1	-44.9 ± 1.1	26.2 ± 0.5	32.7 ± 0.4	34.6 ± 0.6^{A}
23_{Sc}-9_{Ho} (D)	2.3 ± 0.2	25.4 ± 2.4	6.3 ± 0.4^{F}	4.9 ± 0.5	4.2 ± 0.3	$-41.2 \pm 0.7^{A",C",D",F"}$	$25.7 \pm 0.2^{E",G"}$	32.5 ± 0.4	34.3 ± 0.6
23_{Sc}-18_{Ho} (E)	3.2 ± 0.1	27.6 ± 1	6.6 ± 0.2^{F}	5.3 ± 0.2	4.3 ± 0.2	$-35.3 \pm 0.8^{A",C",D",F"}$	$25.1 \pm 0.1^{A"}$	32.2 ± 0.4	34.1 ± 0.6
42_{Sc}-0_{Ho} (F)	$3.24.9 \pm 0.1$	26.4 ± 0.3	7.8 ± 0.4	6.1 ± 0.3	4.9 ± 0.3	-44.7 ± 1.1	26.1 ± 0.6	32.8 ± 0.6	34.6 ± 0.6
42_{Sc}-18_{Ho} (G)	$3.34.9 \pm 0.1$	27.0 ± 1.1	7.3 ± 0.5	5.7 ± 0.3	4.5 ± 0.2	$-36.3 \pm 1.1^{A",C",D",F"}$	$25.2 \pm 0.3^{A"}$	32.5 ± 0.5	34 ± 0.2^{A}

8 Multiphase Structure Results Experiment 1

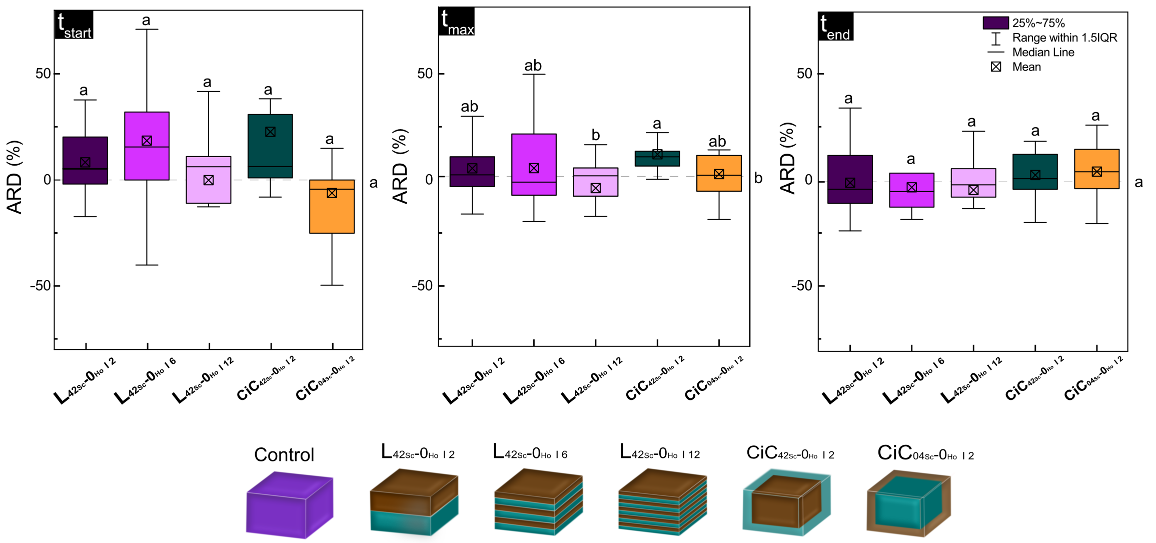

Supplementary Figure S 4. Average relative difference of sweetness perception from control (ARD) for experimental set 1, evaluated at the time points $_{start}$, t_{max} and t_{end}. ARD values with different subscripts are statistically distinguishable ($p < 0.05$).

9 Sucrose delivery quantification

In this test design, we specifically investigated the effect of hazelnut oil content on the perceived sweetness of two distinct samples that differed only in their hazelnut oil content. The samples (30 mm x 30 mm x 3 mm) consisted of 70% fat (either pure cocoa butter for sample 1 or an 18 %w/w hazelnut oil blend for sample 2) supplemented with 30% icing sugar. A non-cocoa sample was chosen to isolate and emphasize the effect of hazelnut oil on taste perception. While the sensory evaluation of chocolate masses indicated a significant effect of hazelnut oil, this was particularly evident for masses with intermediate sucrose content (refer to Fig. 2d). However, it remained unclear whether this perceived enhancement was due to cross-modal texture-taste effects (e.g., higher smoothness imparting higher sweetness perception) or to an increased availability of sucrose at the receptor level.

To investigate this, we designed an experiment with 11 panelists, each of whom was instructed to lick four samples (two samples of 1 and 2, presented in a randomized order) for 45 seconds. To standardize conditions, the panelists were instructed to refrain from eating for an hour before the test, and to rinse their tongues with lukewarm water to remove any residues. After the 45-second tasting period, the licked samples were expectorated into a cup lined with cheesecloth, a setup designed to accurately capture the in-mouth aqueous sucrose content. After filtering, the sample was centrifuged for 10 minutes. The supernatant's soluble solids concentration was measured using a refractometer (LLG-uniREFRACTO 5, Lab Logistics Group GmbH, Germany). This approach, adapted from Gunaratne *et al.*[10], also involved documenting salivation rates (saliva per gram of food) and adjusting the Brix measurements for individual saliva composition, acknowledging the significant variability in saliva composition among individuals[11]. A workflow diagram is available in Supplementary Fig. S 5d. In a separate experiment, ten panelists consumed the same set of samples, and were asked to compare the sweetness of the samples in each pair.. The sensory data were analysed with the sensR package[12].

Our findings indicated no significant impact of salivary secretion on the soluble solids content in the chocolate samples ($F(1,33.57) = 3.58$, NS). This observation was supported by the absence of a correlation between soluble solids and salivary excretion, as illustrated in Supplementary Fig. S 5c. Previous studies have suggested that salivary excretion is predominantly influenced by taste rather than mechanical stimulation[13]. In our experiment, the gustatory differences between the samples were likely too subtle to induce noticeable variations in saliva secretion. Despite individual differences in Brix measurements among the panelists (refer to Supplementary Fig. S 5a), a clear trend emerged: samples containing hazelnut oil showed higher soluble solid contents ($F(1,30.29) = 15.31$, $p < 0.001$), averaging more than 2 Brix higher than those made with pure cocoa butter. These results imply that the incorporation of hazelnut oil in chocolate significantly enhances sweetness perception, as all participants perceived the samples with hazelnut oil as sweeter (data not shown). This enhancement is likely due to the increased availability of sucrose, a consequence of the differences in melting enthalpy between hazelnut oil and cocoa butter, which facilitates a more efficient release of sucrose from the fat matrix.

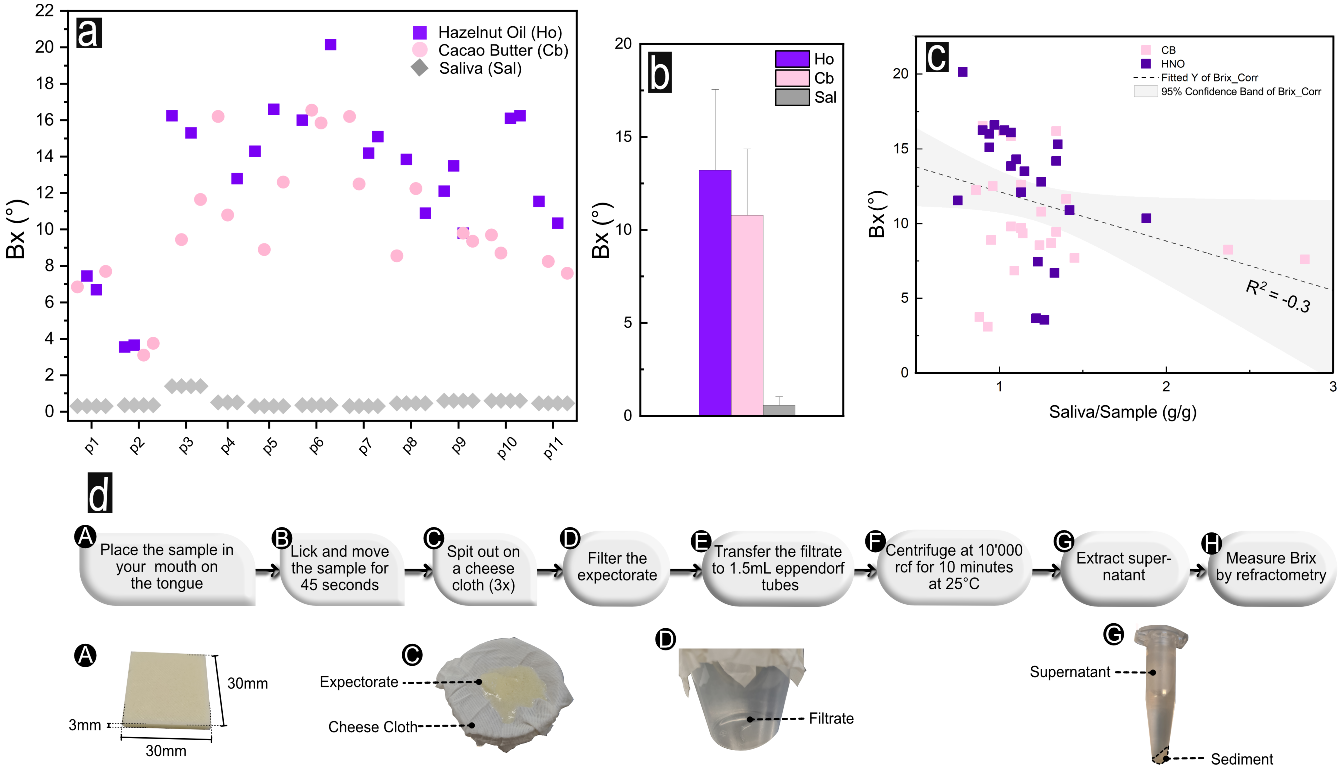

Supplementary Figure S 5. (a) Results of soluble solid contents for samples 1, 2 and pure saliva, separated by panelists. (b) displays the average soluble solid contents for samples 1, 2, as well for saliva, calculated across all participants. (c) Correlation plot between salivary secretion and Brix content (°). (d) outlines the experimental workflow. detailing the process from sample preparation to the collection of expectorates and the subsequent measurement of their Brix content.

10 Tribology measurements

All chocolate samples underwent analysis using an Anton Paar MCR702 MultiDrive rheometer, equipped with a Ball on Three Pins (BTP) tribology cell and a CTD180 temperature control element (all Anton Paar GmbH, Austria). A 0.127 m diameter glass ball (BC12.7, Anton Paar GmbH, Austria) and three smooth, hydrophobic polydimethylsiloxane (PDMS) pins (Sylgard^{TM} 184 silicone elastomer, base and curing agents, Dow Corning, USA) served as tribology surfaces. The melted chocolate samples were carefully transferred into the tribology cell using a metal spoon.

Measurements began after 30 minutes of temperature equilibration at 35 °C. All chocolate masses were analyzed under a normal force of $F_{N,tribo}$ = 1 N. The friction force was measured against sliding velocity, ranging from 10^{-8} m/s to 1 m/s. Pure cocoa butter was assessed as a reference. Each sample underwent ten consecutive measurements with alternating increasing and decreasing speed ramps. The initial two repeats were excluded due to substantial deviation from subsequent runs. The average and standard deviation from the next four runs were calculated for both ramp up and ramp down and were used to derive the Stribeck curve. The increasing velocity ramp curves were used for evaluation. Post-measurement, each sample was removed and the upper and lower parts of the cell were thoroughly cleaned with hot water and a standard cleaning agent.

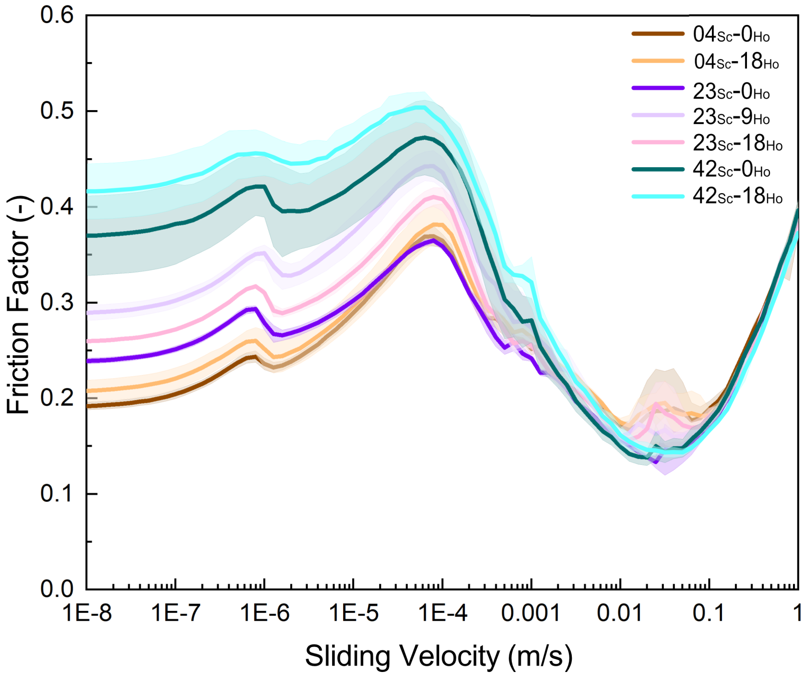

Supplementary Figure S 6. Stribeck curves of the chocolate masses 04_{Sc}-0_{Ho}, 04_{Sc}-18_{Ho}, 23_{Sc}-0_{Ho}, 23_{Sc}-18_{Ho}, 42_{Sc}-0_{Ho}, and 42_{Sc}-18_{Ho}, obtained at 35 °C and at a normal force of $F_{N,tribo}$ = 1 N with a BTP geometry.

11 Panel performance

Supplementary Table S 8. Panel performance | single masses: Discrimination and agreement were based on p-values, and repeatability was based on the mean squared error (MSE). The model considered was: attribute $\sim$ product+panelist+product:panelist. For all panel descriptors, panel performance was categorized into four levels: Good (for discrimination $p \leqslant 0.05$, for agreement $p \geqslant 0.15$), borderline (for discrimination $p \leqslant 0.10$, for agreement $p = 0.10 - 0.15$), poor (for discrimination $p \leqslant 0.15$, for agreement $p = 0.05 - 0.10$), and bad (for discrimination $p > 0.15$ and panel agreement $p \leqslant 0.05$).

	Discrimination	Agreement	Repeatability
Sweetness	< 0.001	0.509	8.51
Bitterness	< 0.001	0.656	6.93
Cocoa	0.068	0.125	5.91
Smooth	0.068	0.003	6

Supplementary Table S 9. Panel performance | multiphase structures of experiment 1: Discrimination and agreement were based on p-values, and repeatability was based on MSE. The model considered was: attribute $\sim$ product+panelist+product:panelist. For all panel descriptors, panel performance was categorized into four levels: Good (for discrimination $p \leqslant 0.05$, for agreement $p \geqslant 0.15$), borderline (for discrimination $p \leqslant 0.10$, for agreement $p = 0.10 - 0.15$), poor (for discrimination $p \leqslant 0.15$, for agreement $p = 0.05 - 0.10$), and bad (for discrimination $p > 0.15$ and panel agreement $p \leqslant 0.05$).

	Discrimination	Agreement	Repeatability
Initial Sweetness	0.013	0.654	0.628
Maximum Sweetness	0.023	0.146	0.969
Last Sweetness	0.299	0.358	0.979

Supplementary Table S 10. Panel performance | multiphase structures of experiment 2: Discrimination and agreement was based on p-values, and repeatability was based on MSE. The model considered was: attribute $\sim$ product+panelist+product:panelist. For all panel descriptors, panel performance was categorized in four levels: Good (for discrimination $p \leqslant 0.05$, for agreement $p \geqslant 0.15$), borderline (for discrimination $p \leqslant 0.10$, for agreement $p = 0.10 - 0.15$), poor (for discrimination $p \leqslant 0.15$, for agreement $p = 0.05 - 0.10$), and bad (for discrimination $p > 0.15$ and panel agreement $p \leqslant 0.05$).

	Discrimination	Agreement	Repeatability
Maximum sweetness	0.03	0.166	0.666
Maximum bitterness	0.375	0.533	0.944
Maximum smoothness	0.043	0.482	0.003
Overall sweetness	0.048	0.224	0.744
Overall smoothness	0.071	0.547	0.059

Supplementary Table S 11. Panel performance | multiphase structures of experiment 2, specifically time-intensity: For analysis, the normalized start, maximum and final sweetness were compared for panel performance. Discrimination and agreement were based on p-values, and repeatability was based on MSE. The model considered was: attribute $\sim$ product+panelist+product:panelist. For all panel descriptors, panel performance was categorized into four levels: Good (for discrimination $p \leqslant 0.05$, for agreement $p \geqslant 0.15$), borderline (for discrimination $p \leqslant 0.10$, for agreement $p = 0.10 - 0.15$), poor (for discrimination $p \leqslant 0.15$, for agreement $p = 0.05 - 0.10$), and bad (for discrimination $p > 0.15$ and panel agreement $p \leqslant 0.05$).

	Discrimination	Agreement	Repeatability
$I_{\text{start-norm}}$	< 0.001	0.108	0.22
$I_{\text{max-norm}}$	< 0.001	0.115	0.223
$I_{\text{last-norm}}$	< 0.001	0.118	0.226

References

1. Karalus, M., Pontet, C. & Vickers, Z. Experimentally Created Intensity Scales for the Five Basic Tastes: Sweet, Sour, Salty, Bitter and Umami [Poster Presentation]. Tech. Rep. 1, University of Minnesota Department of Food Science and Nutrition (2016).
2. Overbosch, P., Enden, J. d. & Keur, B. M. An improved method for measuring perceived intensity/time relationships in human taste and smell. *Chem. Senses* **11**, 331–338, DOI: 10.1093/chemse/11.3.331 (1986).
3. Liu, Y. H. & Macfie, H. J. Methods for averaging time - intensity curves. *Chem. Senses* **15**, 471–484, DOI: 10.1093/chemse/15.4.471 (1990).
4. Hothorn, T., Hornik, K., van de Wiel, M. A. & Zeileis, A. Implementing a class of permutation tests: The {coin} package. *J. Stat. Softw.* **28**, 1–23, DOI: 10.18637/jss.v028.i08 (2008).
5. Bates, D., Mächler, M., Bolker, B. & Walker, S. Fitting Linear Mixed-Effects Models Using {lme4}. *J. Stat. Softw.* **67**, 1–48, DOI: 10.18637/jss.v067.i01 (2015).
6. Hartig, F. DHARMa: Residual Diagnostics for Hierarchical (Multi-Level / Mixed) Regression Models (2022).
7. Lenth, R. V. emmeans: Estimated Marginal Means, aka Least-Squares Means (2023).
8. de Mendiburu, F. agricolae: Statistical Procedures for Agricultural Research (2023).
9. Patil, I. Visualizations with statistical details: The {'ggstatsplot'} approach. *J. Open Source Softw.* **6**, 3167, DOI: 10.21105/joss.03167 (2021).
10. Gunaratne, T. M. *et al.* Physiological responses to basic tastes for sensory evaluation of chocolate using biometric techniques. *Foods* **8**, 1–16, DOI: 10.3390/foods8070243 (2019).
11. Mosca, A. C. & Chen, J. Food-saliva interactions: Mechanisms and implications. *Trends Food Sci. Technol.* **66**, 125–134, DOI: 10.1016/j.tifs.2017.06.005 (2017).
12. Christensen, R. H. B. & Brockhoff, P. B. sensR—An R-package for sensory discrimination (2020).
13. Gavião, M. B. D., Engelen, L. & Van Der Bilt, A. Chewing behavior and salivary secretion. *Eur. J. Oral Sci.* **112**, 19–24, DOI: 10.1111/j.0909-8836.2004.00105.x (2004).
